# *Drosophila* Insulin receptor regulates the persistence of injury-induced nociceptive sensitization

**DOI:** 10.1242/dmm.034231

**Published:** 2018-05-10

**Authors:** Seol Hee Im, Atit A. Patel, Daniel N. Cox, Michael J. Galko

**Affiliations:** 1 Department of Genetics, University of Texas MD Anderson Cancer Center, 1515 Holcombe Boulevard, Houston, TX 77030, USA; 2 Neuroscience Institute, Georgia State University, P.O. Box 5030, Atlanta, GA 30303, USA; 3 Genetics and Epigenetics Graduate Program, University of Texas Graduate School of Biomedical Sciences, 6767 Bertner Avenue, Houston, TX 77030, USA

**Keywords:** Nociceptive sensitization, Insulin receptor, Diabetes, Sensory neurons, Hyperalgesia, *Drosophila*

## Abstract

Diabetes-associated nociceptive hypersensitivity affects diabetic patients with hard-to-treat chronic pain. Because multiple tissues are affected by systemic alterations in insulin signaling, the functional locus of insulin signaling in diabetes-associated hypersensitivity remains obscure. Here, we used *Drosophila* nociception/nociceptive sensitization assays to investigate the role of Insulin receptor (Insulin-like receptor, InR) in nociceptive hypersensitivity. *InR* mutant larvae exhibited mostly normal baseline thermal nociception (absence of injury) and normal acute thermal hypersensitivity following UV-induced injury. However, their acute thermal hypersensitivity persists and fails to return to baseline, unlike in controls. Remarkably, injury-induced persistent hypersensitivity is also observed in larvae that exhibit either type 1 or type 2 diabetes. Cell type-specific genetic analysis indicates that *InR* function is required in multidendritic sensory neurons including nociceptive class IV neurons. In these same nociceptive sensory neurons, only modest changes in dendritic morphology were observed in the *InR^RNAi^*-expressing and diabetic larvae. At the cellular level, *InR*-deficient nociceptive sensory neurons show elevated calcium responses after injury. Sensory neuron-specific expression of InR rescues the persistent thermal hypersensitivity of *InR* mutants and constitutive activation of InR in sensory neurons ameliorates the hypersensitivity observed with a type 2-like diabetic state. Our results suggest that a sensory neuron-specific function of InR regulates the persistence of injury-associated hypersensitivity. It is likely that this new system will be an informative genetically tractable model of diabetes-associated hypersensitivity.

## Abstract (precis)

**Summary:** *Drosophila* insulin signaling is required within nociceptive sensory neurons to regulate the persistence of thermal pain sensitization. We describe a model that could be useful to dissect diabetes-induced pain syndromes.

## INTRODUCTION

*Drosophila* has emerged as a useful system for the study of insulin signaling/diabetes and nociception. With respect to insulin signaling, flies have a canonical Insulin receptor (Insulin-like receptor, InR) (Fernandez et al., 1995), a collection of *Drosophila* insulin-like peptides (Ilps) (Ikeya et al., 2002) manufactured by insulin-producing cells (IPCs) in the brain, and a downstream signal transduction cascade consisting of conserved components (Teleman, 2010). Dysregulation of Ilp production leads to a type 1-like diabetic state in *Drosophila* larvae (Rulifson et al., 2002), while a high-sugar diet leads to insulin resistance and a type 2-like diabetic state (Morris et al., 2012; Musselman et al., 2011; Skorupa et al., 2008). Together, insulin signaling and diabetic states in *Drosophila* regulate systemic glucose metabolism and organ-specific metabolic programs that impact muscle/cardiac function (Demontis and Perrimon, 2010; Na et al., 2013) and immunity (Musselman et al., 2017). However, whether diabetic larvae exhibit the types of sensory phenotypes often associated with diabetic patients remains unclear.

*Drosophila* is also a powerful model for nociception and nociceptive sensitization (Himmel et al.; Im and Galko, 2012). Many of the essential cell types and molecular players are conserved across phyla. At the cellular level, responses to noxious heat and noxious mechanical stimuli in larvae are detected by class IV multidendritic (md) neurons (Hwang et al., 2007), the dendrites of which tile over the barrier epidermis (Grueber et al., 2002) and the axons of which connect to a variety of functionally important second-order neurons in the larval ventral nerve cord (Hu et al., 2017; Yoshino et al., 2017; Ohyama et al., 2015). A number of conserved signaling pathways regulate tissue damage-induced nociceptive sensitization (Gold and Gebhart, 2010). In *Drosophila* larvae, these include Tumor necrosis factor (TNF; Egr) (Babcock et al., 2009), Hedgehog (Hh) (Babcock et al., 2011) and Substance P/Tachykinin (Tk) (Im et al., 2015). Whether baseline nociception (in the absence of injury) or injury-induced nociceptive sensitization is altered by disease-like states, such as diabetes, remains an open question in *Drosophila*.

Patients with diabetes often experience discomfiting alterations in sensory perception as the disease progresses (Veves et al., 2008). These changes often begin with nociceptive hypersensitivity to temperature and touch before progressing to numbness or hyposensitivity. There is substantial debate about the etiology of these diabetes-induced sensory alterations (Obrosova, 2009; Zochodne, 2016), including their relation to systemic glucose levels, diabetes-induced vascular changes, peripheral neurodegeneration or neuronal functions of insulin signaling (Gralle, 2017; Grote and Wright, 2016). More recently, evidence has emerged of sensory neuron intrinsic factors (Tsantoulas et al., 2017) and glucose toxicity affecting sensory perception of painful stimuli (Bierhaus et al., 2012; Orestes et al., 2013). Most vertebrate experimental models of the painful diabetic neuropathy involve either systemic pharmacological treatments or whole-animal genetic alterations (Obrosova, 2009). One unaddressed question is the relative contribution of various tissues, including peripheral sensory neurons, to painful sensory alterations. To date, there have been no models of diabetes-associated nociceptive changes that employ highly genetically tractable organisms such as *Drosophila*.

## RESULTS

### *InR* mutant larvae exhibit persistent thermal hyperalgesia

**Fig. 1.**
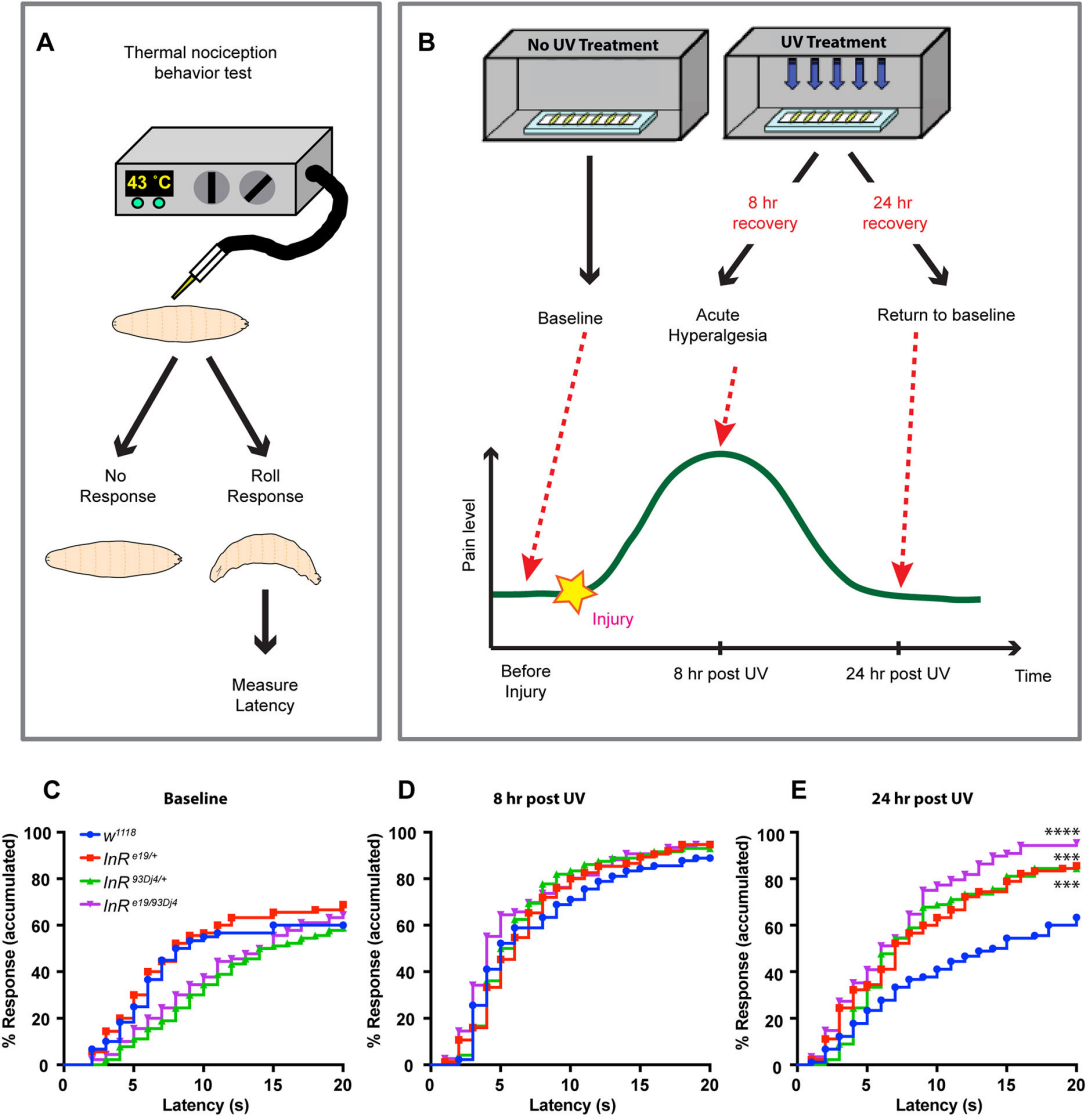
***InR* mutant larvae exhibit persistent thermal hyperalgesia.** (A,B) Schematics of the nociception (A) and persistent nociceptive sensitization (B) assays. (C-E) Quantitation of nociceptive behavioral responses to thermal stimulation at 43°C of *InR* mutant larvae. *w^1118^* control larvae, two heterozygous hypomorphic alleles and a transheterzygous allelic combination of *InR* were tested: *InR^e19^*^/+^, *InR^93Dj4^*^/+^, *InR^e19^*^/*93Dj4*^. Baseline responses without UV tissue damage (*n*=60 for *w^1118^*, *n*=90 for others) (C), thermal sensitivity at 8 h post-UV (*n*=90 for *w^1118^*, *n*=80 for *InR^e19^*^/+^, *n*=88 for *InR^93Dj4^*^/+^, *n*=76 for *InR^e19^*^/*93Dj4*^) (D), thermal sensitivity at 24 h post-UV (*n*=88 for *InR^e19^*^/*93Dj4*^, *n*=90 for others) (E). Statistical significance was determined by the Log-rank test. ***P<0.001, ****P<0.0001.

To explore the possibility that larvae with alterations in insulin signaling might exhibit nociceptive phenotypes, we first tested whether *InR* mutant larvae exhibited changes in baseline thermal nociception and thermal hyperalgesia (increased sensitivity to noxious thermal stimuli), using assays standard in the field (Chattopadhyay et al., 2012) (Fig. 1A,B). Because homozygous *InR* loss-of-function mutants are larval lethal (Chen et al., 1996), we tested larvae heterozygous for two hypomorphic alleles of *InR* (*InR^e19^*, *InR^93Dj4^*), and larvae transheterozygous for the two alleles (*InR^e19^*^/*93Dj4*^) (Tatar et al., 2001). These larvae had a normal number of responders during baseline thermal nociception in response to a noxious stimulus (43°C) in the absence of injury (Fig. 1C), although there was a difference in the average latency of the responders for the *InR^93Dj4/+^* and *InR^e19^*^/*93Dj4*^ alleles (Fig. S1). After UV-induced tissue injury (Babcock et al., 2009), both control and *InR* mutant larvae showed a normal acute thermal hyperalgesia response at 8 h post-injury when tested at this same temperature (Fig. 1D). However, in *InR* mutants, this acute sensitization failed to resolve over the normal time course (Fig. 1E), and continued as persistent thermal hyperalgesia at a time (24 h post-injury) when acute sensitization has resolved in controls. The persistent thermal hyperalgesia phenotype is significant in *InR* heterozygotes and is more severe in the transheterozygous larvae (Fig. 1E). Therefore, whole-animal *InR* mutant larvae exhibit persistent thermal hypersensitivity, a phenotype reminiscent of the early phase of painful diabetic neuropathy.

### Type 1 diabetic larvae exhibit persistent thermal hyperalgesia after injury

**Fig. 2.**
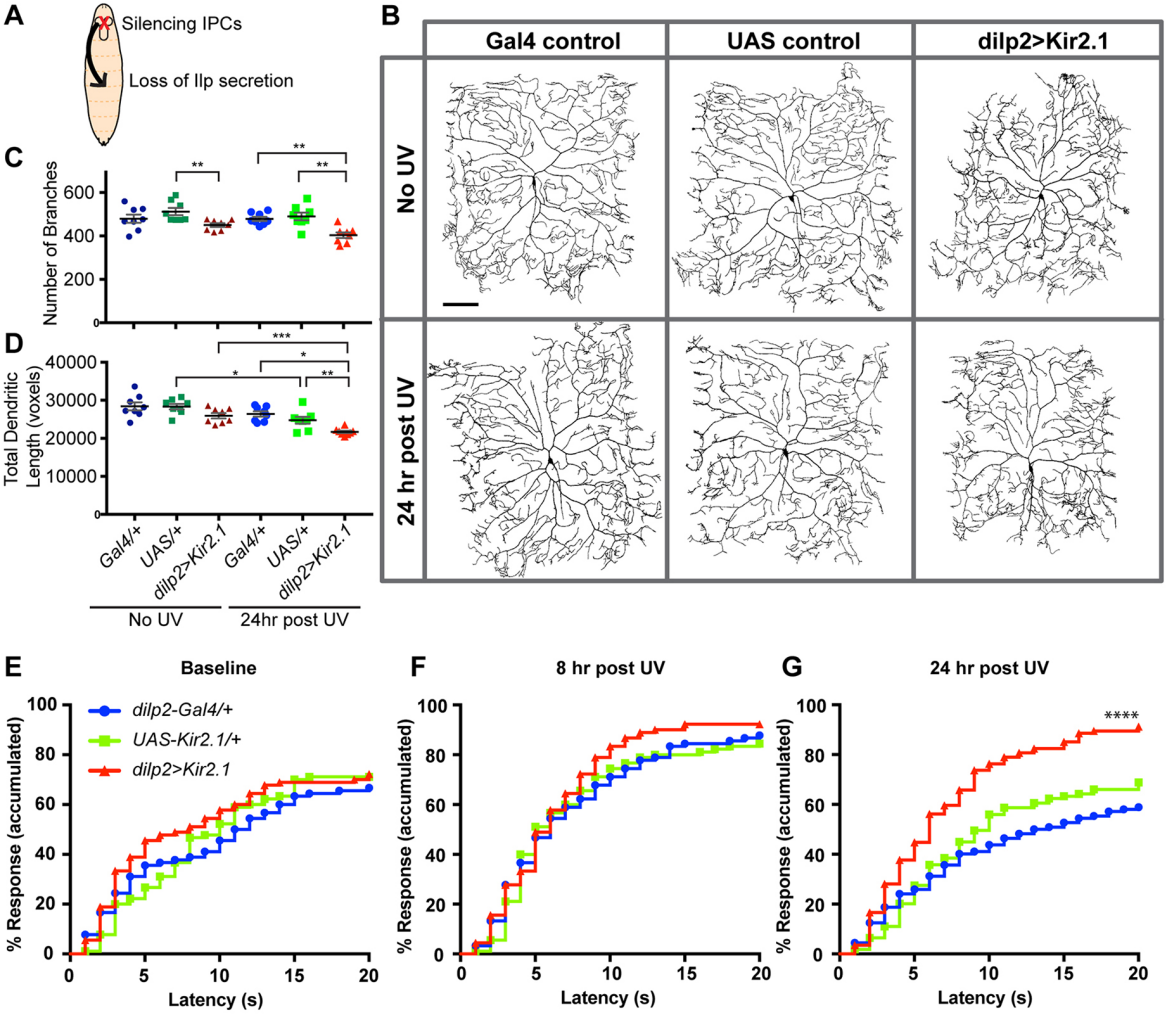
**A type 1 diabetes-like state induces persistent thermal hyperalgesia in *Drosophila* larvae.** (A) Schematic of the genetic manipulation that induces a type 1 diabetes-like state in *Drosophila* larvae by silencing IPCs. (B) Representative *in vivo* confocal images of class IV md neuron dendritic morphology in controls and in larvae exhibiting a type 1 diabetes-like state±UV irradiation. In all panels, dendritic morphology was visualized using a *ppk-CD4::tdTomato* transgene. Controls: *dilp2-Gal4* alone and *UAS-Kir2.1* alone. Type 1 Diabetes: *dilp2-Gal4*>*UAS-Kir2.1*. (C,D) Quantitative dendritic morphology analysis measuring number of branches (C) and total dendritic length (D) presented as mean±s.e.m. *n*=8 neurons. Statistical significance was determined by one-way ANOVA with Bonferroni multiple comparison post hoc test. (E-G) Quantitation of nociceptive behavioral responses to thermal stimulation (43°C) in control larvae and when IPCs were silenced genetically. In all behavioral analyses, accumulated total responses were plotted as a function of latency to aversive withdrawal. Baseline behavioral responses in the absence of UV irradiation (E), thermal sensitivity at 8 h post-UV (F), thermal sensitivity at 24 h post-UV (G). *n*=90 larvae tested for each condition. *P<0.05, **P<0.01, ***P<0.001, ****P<0.0001.

To determine whether the persistent thermal hyperalgesia observed in *InR* mutant larvae might be related to diabetes-induced sensory changes, we utilized both type 1 and type 2 diabetes models (Musselman et al., 2011; Rulifson et al., 2002). To create a type 1 diabetes-like state (Fig. 2A), we silenced insulin-producing cells (IPCs) by expressing an inward rectifying potassium channel (Kir2.1) using the *dilp2* (*Ilp2*)*-Gal4* driver, which is specific for IPCs (Rulifson et al., 2002). Expression of Kir2.1 in these cells results in a lack of circulating *Drosophila* insulin-like peptides 2, 3 and 5 (Ilp2, 3, 5) (Park et al., 2014). Morphologically, silencing of IPCs in the absence of UV-induced injury did not significantly reduce the number of branches or total dendritic length of class IV neurons compared with Gal4 alone controls, although it was significant against UAS alone controls. Type 1 diabetic larvae when UV irradiated exhibited a reduction in number of branches and total dendritic length compared with irradiated Gal4 and UAS alone controls (Fig. 2B-D). Behaviorally, the baseline (absence of injury) nociceptive sensitivity of type 1 diabetic larvae at 43°C was similar to that of the Gal4 and UAS alone control larvae (Fig. 2E). Similarly, both controls and type 1 diabetic larvae showed similar responses to a 43°C probe at the peak hyperalgesia time point (Fig. 2F) following UV-induced tissue injury. By contrast, we found that the type 1 diabetes-like state resulted in persistent hypersensitization. Control larvae invariably resolved their thermal hyperalgesia by 24 h following injury (Fig. 2G). Type 1 diabetic larvae, by contrast, still exhibited thermal hyperalgesia at this time (Fig. 2G). Therefore, a type 1 diabetes-like condition results in injury-induced persistent nociceptive hypersensitivity with no alteration in the baseline sensitivity or acute thermal hyperalgesia, similar to what is observed in *InR* mutants.

### Type 2 diabetic larvae exhibit persistent thermal hyperalgesia after injury

**Fig. 3.**
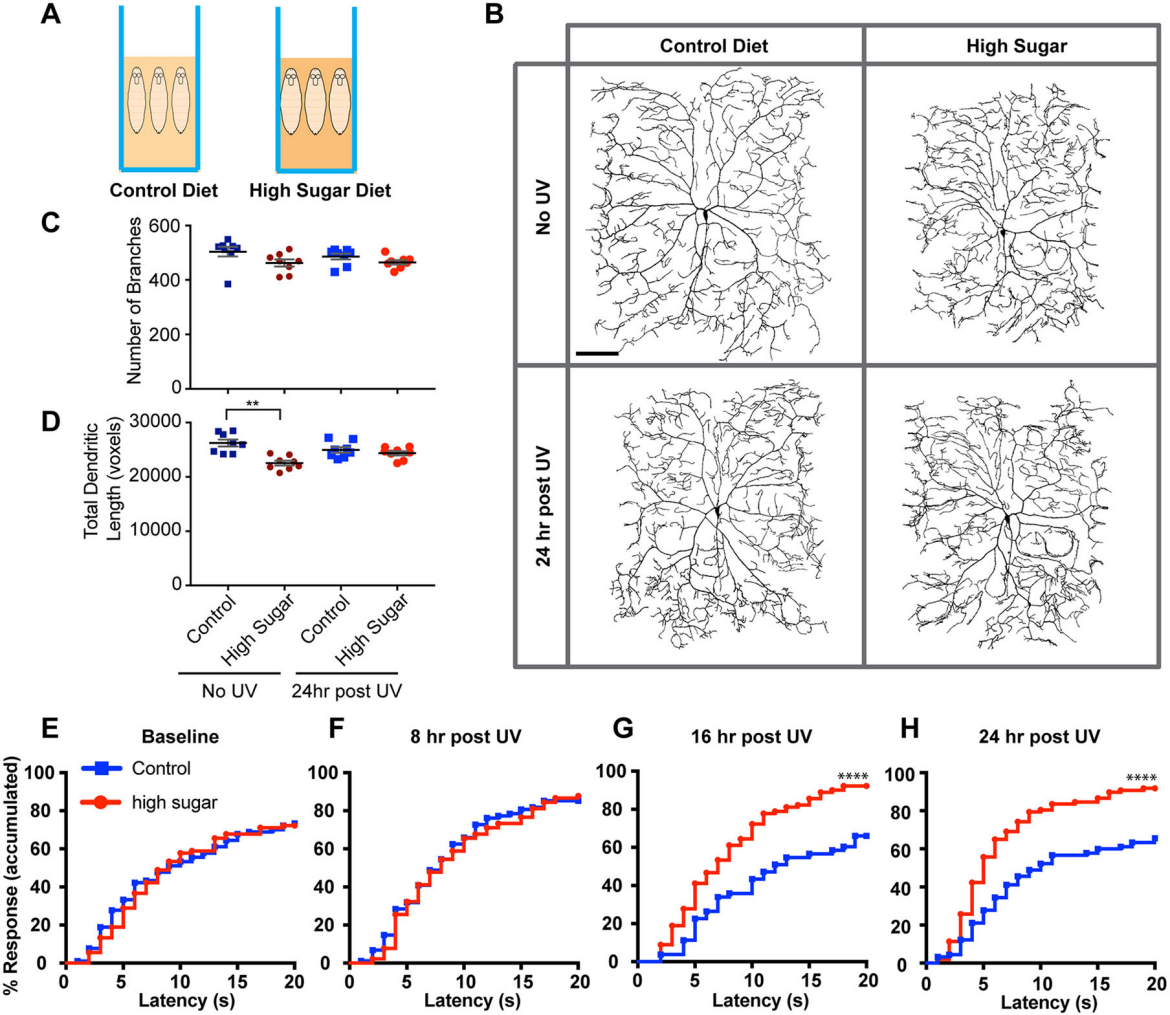
**A type 2 diabetes-like state induces persistent thermal hyperalgesia in *Drosophila* larvae.** (A) Schematic of the diet condition (high sugar) that induces a type 2 diabetes-like state in exposed larvae. (B) Representative *in vivo* confocal images of class IV md neuron dendritic morphology in controls (normal diet) and in larvae exhibiting a type 2 diabetes-like state (high-sugar diet)±UV irradiation. Dendritic morphology was visualized using a *ppk-CD4::tdTomato* transgene. (C,D) Quantitative dendritic morphology analysis measuring number of branches (C) and total dendritic length (D) presented as mean±s.e.m. *n*=8 neurons. Statistical significance was determined by one-way ANOVA with Bonferroni multiple comparison post hoc test. (E-H) Quantitation of nociceptive behavioral responses to thermal stimulation (43°C) in control larvae and larvae fed a high-sugar diet. Baseline responses in the absence of UV irradiation (*n*=90 for each condition) (E), thermal sensitivity at 8 h post-UV (*n*=88 for control, *n*=90 for high sugar) (F), thermal sensitivity at 16 h post-UV (*n*=53 for control, *n*=90 for high sugar) (G), thermal sensitivity at 24 h post-UV (*n*=90 for control, *n*=97 for high sugar) (H). **P<0.01, ****P<0.0001.

To model type 2 diabetes (Fig. 3A), we cultured larvae on a high-sugar diet (Musselman et al., 2011). This nutritional regimen results in increased circulating sugar levels, fat accumulation and increased expression of *dilp* (*Ilp*) genes. Morphologically, the type 2 diabetic condition did not affect the number of dendritic branches in class IV md nociceptive neurons (Fig. 3B,C), although it did reduce the total dendritic length (Fig. 3B,D). No significant differences in morphological measures were observed with or without the high-sugar diet after UV-induced tissue injury (Fig. 3B-D).

Behaviorally, type 2 diabetic larvae did not exhibit any defects in baseline thermal nociception (43°C) (Fig. 3E) or in acute thermal hyperalgesia following UV-induced tissue injury (Fig. 3F). To test whether there is developmentally induced shift in the timing or duration of the hyperalgesic peak, we examined larvae 16 h after injury. Control larvae had returned to baseline, as observed before (Babcock et al., 2009), whereas larvae grown on the high-sugar diet remained hypersensitive (Fig. 3G), a condition that persisted 24 h after irradiation (Fig. 3H). Taken together, we found that *Drosophila* larvae with a type 2 diabetes-like state exhibited a highly specific phenotype of persistent thermal hyperalgesia without corresponding defects in baseline thermal nociception or injury-induced acute thermal hyperalgesia.

### Sensory neuron-specific loss of Insulin receptor causes persistent thermal hyperalgesia

The persistent thermal hyperalgesia in *InR* mutants and diabetic larvae suggest that insulin signaling is required to regulate the persistence of acute thermal nociceptive hypersensitivity. We thus asked in which tissue(s) InR function is required for diabetes-associated nociceptive persistence. To address this question, we utilized tissue-specific Gal4 drivers (Table S1) to express a *UAS-RNAi* transgene targeting *InR*. We then tested whether persistent thermal hyperalgesia was observed in progeny larvae expressing the *UAS-InR^RNAi^* transgene in each tissue compared with relevant genetic controls (Gal4 transgenes alone). Larvae with muscle-, fat body- and hemocyte-specific Gal4 expression of *UAS-InR^RNAi^* did not exhibit persistent thermal hyperalgesia (Fig. S2).

**Fig. 4.**
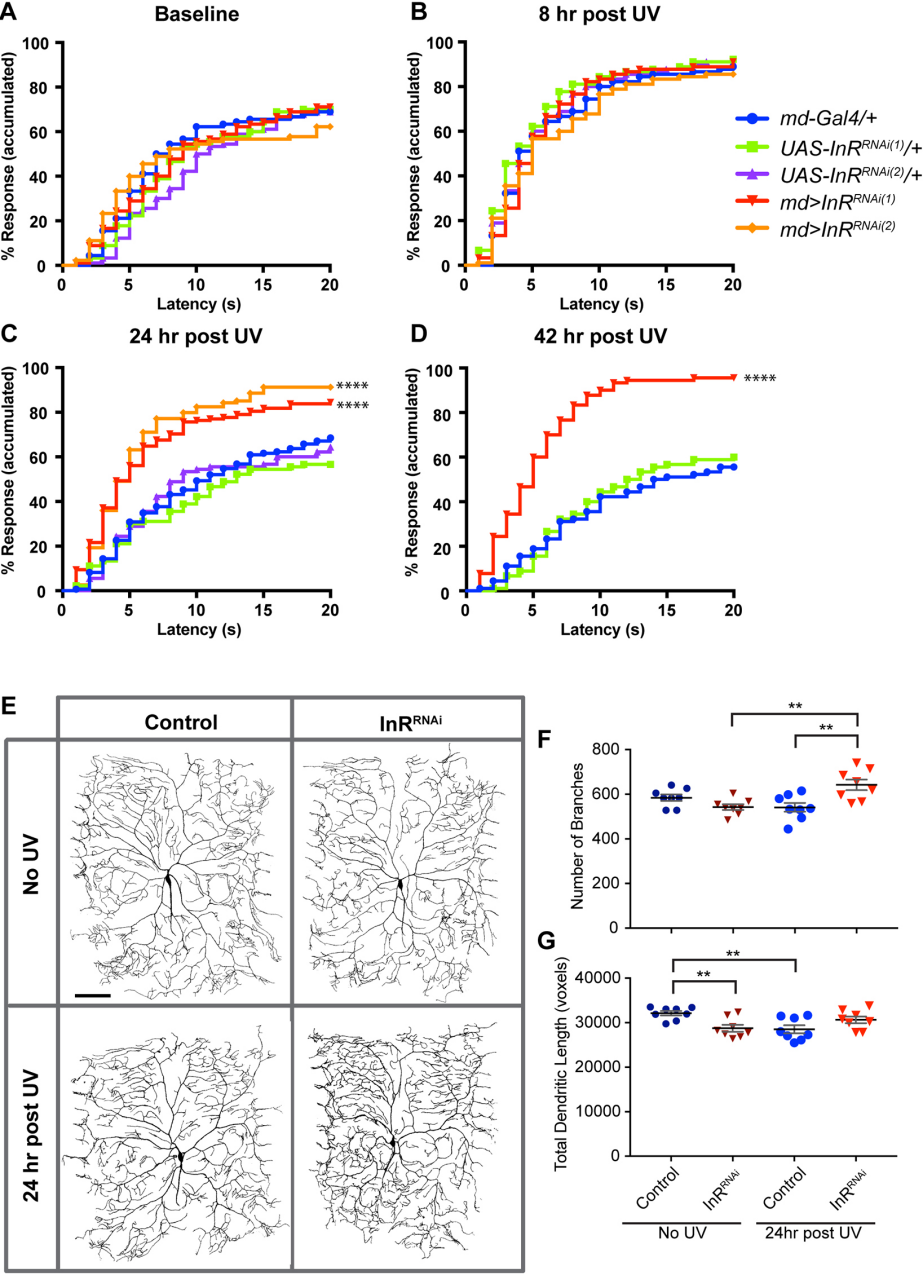
**Sensory neuron-specific interference with InR function causes persistent thermal hyperalgesia.** (A-D) Quantitation of thermal nociceptive behavioral responses (43°C) when *UAS-InR^RNAi^* is expressed in md neurons. *n*=90 for each condition. Baseline responses in the absence of UV irradiation (A), thermal sensitivity at 8 h post-UV (B), thermal sensitivity at 24 h post-UV (C), thermal sensitivity at 42 h post-UV (D). (E) Representative *in vivo* confocal images of class IV md neuron dendritic morphology labeled with *ppk1.9-GAL4,UAS-mCD8::GFP*. Dendritic morphology was compared between control larvae expressing *UAS-Luc^RNAi^* and larvae expressing *UAS-InR^RNAi^*±UV irradiation. (F,G) Quantitative dendritic morphology analysis measuring number of branches (F) and total dendritic length (G) presented as mean±s.e.m. *n*=8 neurons. Statistical significance was determined by one-way ANOVA with Bonferroni multiple comparison post hoc test. **P<0.01.

As InR function was not centered in typical metabolic control tissues, we tested sensory neurons themselves. Expression of *UAS-InR^RNAi^* using a pan-md sensory neuron driver did not cause defects in baseline (Fig. 4A) or acute thermal hyperalgesia (Fig. 4B). By contrast, md neuron expression of *UAS-InR^RNAi^* did result in prolonged thermal hyperalgesia that was apparent 24 h post-injury (Fig. 4C) and worsened throughout the third larval stage (Fig. 4D). These results indicate that perturbing InR function within multidendritic nociceptive sensory neurons, but not within other tissues that typically control metabolic regulation, leads to persistent thermal hyperalgesia following injury.

Specificity to peripheral nociceptive sensory neurons is supported because we also observed persistent thermal hyperalgesia with an independent pan-md driver and a class IV nociceptive sensory neuron md driver (Table S1, Figs S3 and S4). Independent *UAS-InR^RNAi^* transgenes targeting nonoverlapping regions of *InR* (Fig. 4A-C; Fig. S5) gave the same phenotype, as did a *UAS-InR^DN^* transgene (Wu et al., 2005) expressing a dominant negative form of InR (Fig. S6), ruling out RNAi off-target effects. RNAi transgenes targeting other components of the insulin-like signaling (ILS) pathway (*chico*, *Pi3K* genes) also resulted in persistent thermal hyperalgesia (Fig. S7).

Morphologically, md neuron-specific expression of *UAS-InR^RNAi^* did not affect the total number of branches of class IV md neurons under baseline conditions, although it did reduce the total dendritic length (Fig. 4E-G). This pattern of morphological changes is similar to what was observed in the type 2 diabetic condition (Fig. 3B-D). However, with *UAS-InR^RNAi^* expression in md neurons, there was a significant increase in the number of class IV dendritic branches after UV irradiation (Fig. 4E,F) that was not accompanied by a corresponding increase in total dendritic length (Fig. 4E,G). The relationship between the consistent behavioral phenotype and the relatively modest morphological changes across different genotypes and diabetic conditions is discussed further below.

### Persistent thermal hyperalgesia is associated with elevated calcium responses in class IV nociceptive neurons

**Fig. 5.**
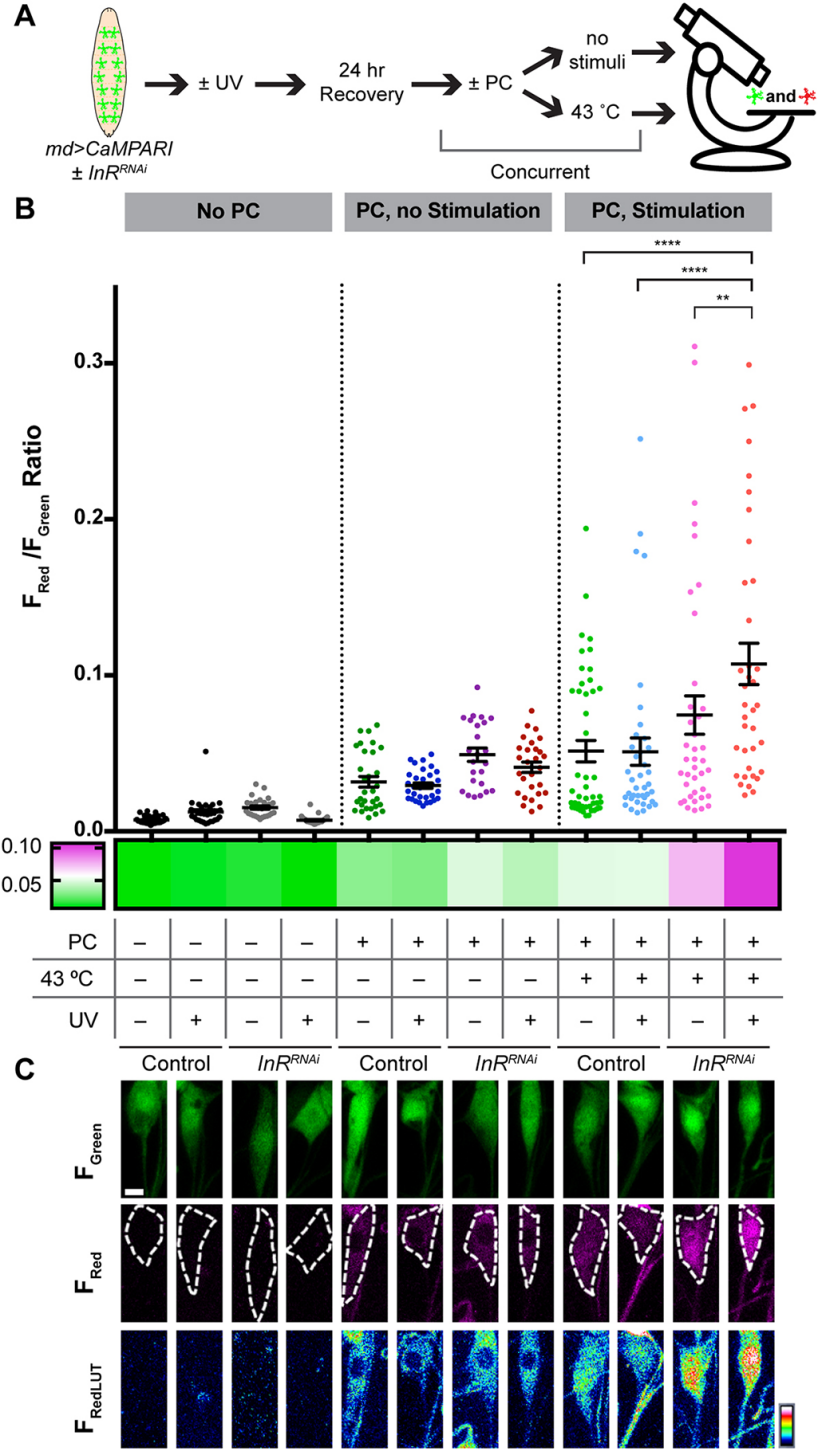
**CaMPARI analysis reveals increased cellular calcium in sensory neurons.** (A) Schematic of CaMPARI experimental outline. (B) Quantitative analysis of CaMPARI responses in class IV md neurons of larvae expressing *UAS-CaMPARI* via *md-Gal4*±*UAS-InR^RNAi^*. The CaMPARI response is calculated as the F_Red_/F_Green_ ratio presented as mean±s.e.m. and is represented graphically, where each measured neuron is represented by a single data point, and also as a heatmap depicting the averaged CaMPARI response. On the heatmap, magenta indicates a higher F_Red_/F_Green_ ratio and green indicates a lower ratio. *n*=24-45 neurons. Statistical significance was determined by one-way ANOVA with Bonferroni multiple comparison post hoc test. A key to relevant experimental variables (PC light, thermal stimulation, UV and genotype) is provided and applies to the quantitative data in B and the micrographs in C. (C) Representative *in vivo* confocal images of class IV md neuronal cell bodies. For each condition the F_Green_, F_Red_ and F_RedLUT_ (a heatmap representation of photoconverted CaMPARI intensity) are shown. **P<0.01, ****P<0.0001.

Is the behavioral hypersensitivity seen upon loss of InR in nociceptive sensory neurons accompanied by cellular level changes in neuronal activity? To assess this, we tried to use GCaMP (Chen et al., 2013) expressed within sensory neurons, but found that the GFP fluorescence was not stable over the duration of the noxious heat exposure (data not shown). As an alternative, we expressed CaMPARI (Fosque et al., 2015), a genetically encoded calcium integrator that undergoes fluorescence conversion from green to red as a function of high intracellular calcium and photoconverting (PC) light, in md neurons with or without *UAS-InR^RNAi^*. Progeny larvae (control and InR loss of function) were mock irradiated or UV irradiated as in our behavioral analysis, and both groups were either challenged with a 43°C heat probe stimulus or not (see Materials and Methods, Fig. 5A for experimental flowchart). In the absence of PC light, the ratio of F_Red_/F_Green_ (CaMPARI response) is low under all conditions tested, as expected (Fig. 5B, no PC). Exposure to PC light led to a slight (but not significant) increase in the CaMPARI response with or without UV irradiation (Fig. 5B, PC, no stimulation). In control larvae, adding a noxious heat stimulus (43°C heat probe) with or without UV-induced injury did not significantly increase the observed CaMPARI response (Fig. 5B, compare PC, no stimulation controls with PC, stimulation controls). For *UAS-InR^RNAi^*-expressing larvae, exposure to a noxious stimulus by itself was not sufficient to cause a significant increase in the CaMPARI response compared with controls (Fig. 5B, PC, stimulation). By contrast, *UAS-InR^RNAi^*-expressing larvae that were UV irradiated and exposed to a noxious heat stimulus showed a significant increase in CaMPARI responses compared with all other relevant conditions (Fig. 5B, PC, stimulation). Representative neuronal cell bodies reflecting the average CaMPARI responses are depicted in Fig. 5C. Together, these results suggest that a significant increase in neuronal calcium is apparent when UV-induced tissue injury is combined with heat stimulation. Similar to our behavioral results, this increase at 24 h postirradiation is only seen when InR function is reduced in md neurons.

### Constitutive activation of InR causes hyposensitivity during the acute phase

**Fig. 6.**
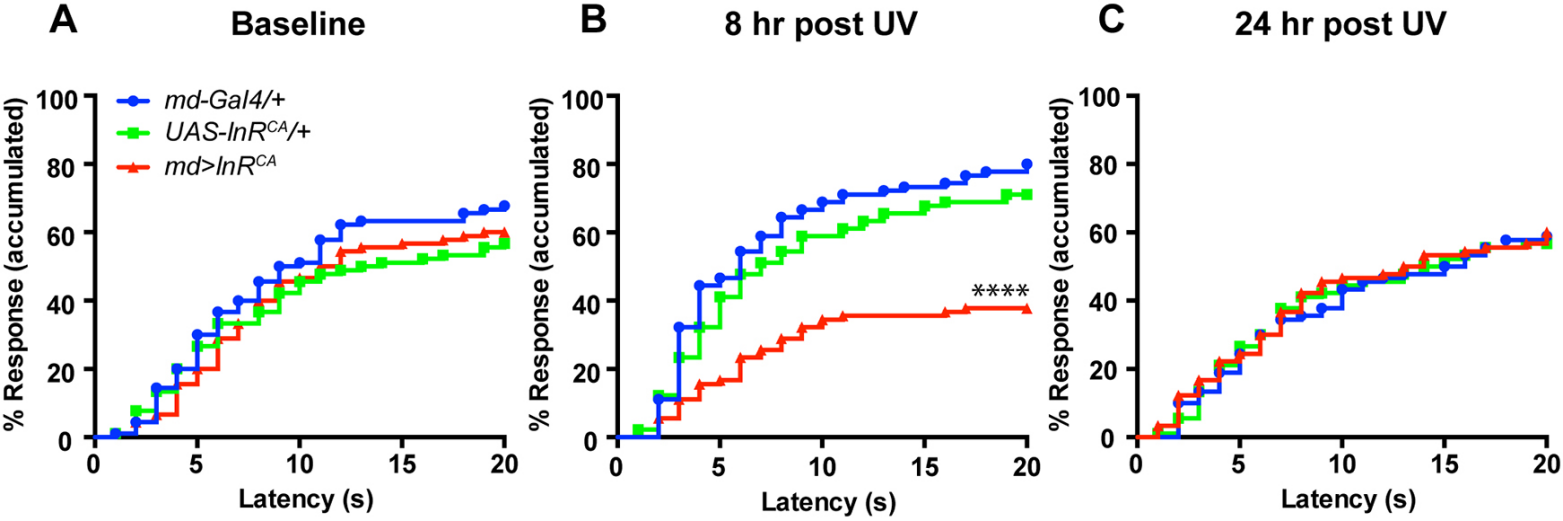
**Constitutive activation of InR causes hyposensitivity during the acute phase.** (A-C) Quantitation of thermal nociceptive behavioral responses (43°C) when *UAS-InR^CA^* is expressed in md neurons versus Gal4 and UAS alone controls. Baseline responses in the absence of UV irradiation (A), thermal sensitivity at 8 h post-UV (B), thermal sensitivity at 24 h post-UV (C). *n*=90 for each condition/genotype. ****P<0.0001.

Our genetic analysis suggests that ILS might be required within md neurons to actively shut off acute thermal sensitization. To test this possibility we overexpressed a constitutive active (CA) form of InR (*UAS-InR^CA^*) (Wang et al., 2008) in md neurons. If ILS is a general regulator of nociceptive sensation, we might expect constitutive activation of this pathway to alter baseline nociception in the absence of injury. This was not observed – *InR^CA^* expression did not alter baseline (no injury) thermal nociception (43°C) (Fig. 6A). However, the acute thermal hyperalgesia, which peaked at 8 h after UV-induced injury in controls, was greatly attenuated in *UAS-InR^CA^*-expressing larvae, even dipping below the normal nociceptive response to the 43°C stimulus (Fig. 6B). When examined at the normal recovery time point (24 h after injury) there were no sensitivity differences between *UAS-InR^CA^*-expressing and relevant control larvae (Fig. 6C). Therefore, constitutive InR activation causes acute hyposensitivity after injury. Baseline nociception was not affected and the injury-induced hyposensitivity resolved with similar kinetics compared with the normal injury-induced hypersensitivity.

### Multidendritic neuron-specific restoration of ILS rescues persistent nociceptive hypersensitivity

**Fig. 7.**
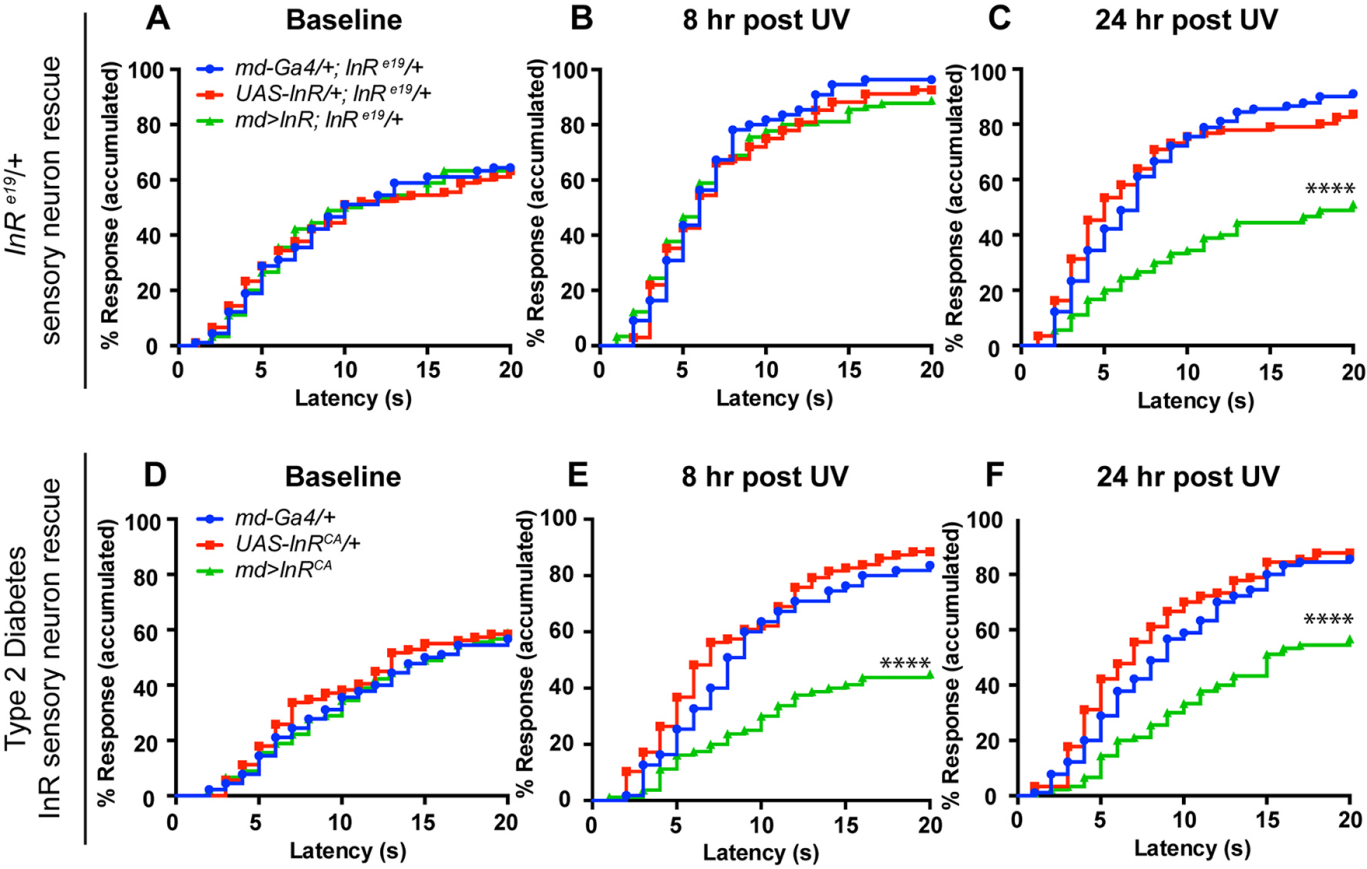
**Expression of InR in md neurons rescues the persistent thermal hyperalgesia of *InR* mutants and type 2 diabetic larvae.** (A-C) Quantitation of nociceptive behavioral responses (43°C) when *UAS-InR* is expressed in md sensory neurons in the heterozygous *InR* mutant background. Baseline responses without UV irradiation (A), UV-induced acute hyperalgesia (8 h post-UV) (B), UV-induced persistent hyperalgesia (24 h post-UV) (C) (*n*=90 for each condition/genotype). (D-F) Quantitation of nociceptive behavior responses (43°C) when *UAS-InR^CA^* is expressed in md sensory neurons in the high-sugar fed larvae. Baseline responses without UV irradiation (*n*=90) (D), UV-induced acute hyperalgesia (8 h post-UV) (*n*=55 for Gal4 alone, *n*=87 for UAS alone, *n*=80 for *md*>*InR^CA^*) (E), UV-induced persistent hyperalgesia (24 h post-UV) (*n*=90) (F). ****P<0.0001.

The finding that md neuron-specific loss causes persistent thermal hyperalgesia suggests that normal ILS is required in md neurons to successfully turn off injury-induced acute sensitization. The dampening of acute thermal hyperalgesia upon constitutive activation of ILS supports this idea. To further test this hypothesis we attempted to rescue ILS function in md sensory neurons both in *InR* mutants and in type 2 diabetic larvae. We expressed a *UAS-InR* transgene specifically in md neurons in a heterozygous *InR^e19^* background and tested the resulting larvae for thermal sensitization versus relevant genetic controls. There were no differences in baseline thermal nociception (Fig. 7A) or acute thermal hyperalgesia (Fig. 7B) between the rescued larvae and the controls. However, at the recovery time point, we found that larvae expressing *UAS-InR* in their nociceptive sensory neurons showed a normal recovery, whereas control larvae (Gal4 or UAS transgene alone) lacking InR expression still showed persistent thermal hyperalgesia (Fig. 7C). We also found that constitutively activating InR in nociceptive sensory neurons of type 2 diabetic larvae did not affect baseline nociception (Fig. 7D), but caused hypoalgesia at the acute time point (Fig. 7E) coupled with a normal recovery to baseline at 24 h (Fig. 7F). Together, these results support the hypothesis that the function(s) of InR relevant to regulating nociceptive sensitivity following injury or induction of a diabetic state lie mainly within nociceptive sensory neurons.

## DISCUSSION

**Fig. 8.**
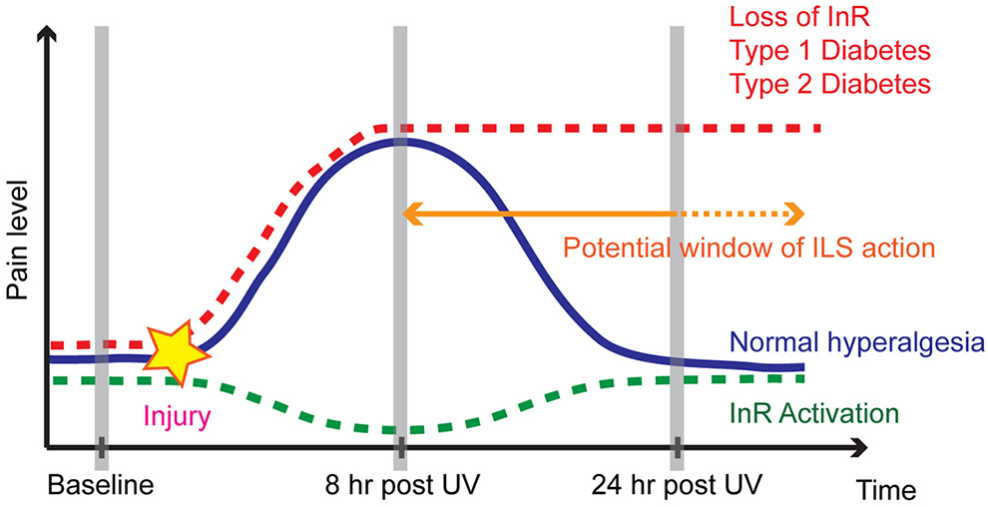
**Graphical representation of pain levels versus time postinjury, annotated across control, persistently hypersensitive genotypes and diabetic conditions, and upon constitutive activation of InR**. Control, blue solid line; persistently hypersensitive genotypes and diabetic conditions, red dashed line; constitutive activation of InR, green dashed line. Landmark time points (gray vertical bars) and the likely window of ILS activity in nociceptive sensory neurons (orange arrow/text) are indicated.

Our results suggest that InR function in nociceptive md sensory neurons is important to regulate the persistence of injury-induced nociceptive sensitization (Fig. 8). *InR* mutants exhibit a highly specific phenotype whereby baseline nociception and the normal acute nociceptive sensitization response are unaffected. In *InR* mutant larvae, however, the acute response does not resolve back to baseline, resulting in persistent sensitization. An md neuron-intrinsic function of InR is supported by four lines of evidence: (1) md neuron-specific loss of InR function leads to persistent thermal hyperalgesia; (2) md neuron-specific loss of InR function leads to increased neuronal calcium responses at a time point consistent with behavioral hypersensitivity; (3) md neuron-specific restoration of InR rescues persistent thermal hyperalgesia observed in *InR* mutants; and (4) md neuron-specific constitutive activation of InR dampens the peak of acute injury-induced hyperalgesia. These results suggest that InR function might become active during the recovery phase of sensitization – a conclusion supported by the specific dampening of the acute thermal hyperalgesia response in nociceptive sensory neurons expressing constitutively active InR.

Our results also suggest that the md neuron-specific role of InR might be relevant to diabetes-associated nociceptive phenotypes. First, larvae experiencing both type 1 and type 2 models of diabetes exactly phenocopy the loss of InR in md neurons – persistence of thermal hypersensitivity. Second, md neuron-specific rescue of InR function can ameliorate the persistent thermal hyperalgesia seen in both *InR* mutants and in type 2 diabetic larvae. Below, we discuss the possible implications of these findings in flies and in other models of diabetes-associated pain.

InR has diverse functions in multiple tissues so that each tissue can be responsive to the organism's metabolic state (Demontis and Perrimon, 2010; Wessells et al., 2004). The relevant tissue for the regulation of nociception is not clear. Our data suggest that the activity of InR relevant to nociceptive sensitization is not localized in the major metabolic signaling tissues of the fly larva – fat body, muscle or hemocytes. Rather, InR functions in the very sensory neurons that respond to noxious thermal stimuli. In vertebrates, the insulin receptor is expressed on nociceptive sensory neurons (Sugimoto et al., 2002, 2000), but whether it functions in nociceptive sensory neurons has not been tested to date. Further, the conditional InR knockouts generated to date have not been tested for pain phenotypes (Bruning et al., 2000). However, mouse insulin receptor can regulate synapse number and neuronal plasticity, at least in central nervous system neurons (Chiu et al., 2008; Grillo et al., 2015). Given that nociceptive biology is evolutionarily conserved, our work suggests that it would be interesting to test pain physiology in a nociceptor-specific knockout of the mouse insulin receptor.

With InR loss of function we observe cellular-level changes – increased cellular calcium measured by CaMPARI and modest changes in dendritic morphology – that could conceivably help explain the observed behavioral hypersensitivity. As a genetically encoded calcium integrator, CaMPARI allows for post hoc assessment of neural activation states as a function of stimulus conditions and in combination with genetic perturbations. Previous studies have utilized CaMPARI to measure *in vivo* neuronal activity levels in response to a broad range of sensory stimuli in *Drosophila*, zebrafish and mice (Enjin et al., 2016; Fosque et al., 2015; Turner et al., 2016), as well as in mapping functional synaptic connectivity (Zolnik et al., 2017). In the case of InR function assessed here, CaMPARI analysis indicates increased md neuron function/output at the time when hypersensitivity would have resolved in control larvae. The observed morphology changes, which are modest but significant, are not uniform across diabetic conditions (type 1 and type 2) and genetic manipulations (md neuron-specific expression of *InR^RNAi^*). However, the observed behavioral phenotype – persistent thermal hyperalgesia – is shared across all conditions. For this reason, we suspect that morphological changes at the dendritic level are unlikely to be a major driver of the behavioral phenotype. The morphological changes observed are consistent with the peripheral neuritogenic effects of insulin observed in vertebrate neuronal culture (Fernyhough et al., 1993; Recio-Pinto et al., 1986).

An important question is when ILS is activated following injury. Given that *InR* loss of function results in persistent thermal hyperalgesia following a normal peak of acute hyperalgesia, it seems likely that ILS would be activated after the acute response. The normal thermal nociception baseline and the acute thermal hyposensitivity observed upon constitutive activation of InR are consistent with this idea. There is precedence in *Drosophila* for UV injury increasing ILS in non-neuronal tissues (Karpac et al., 2011). If ILS were activated neuronally after the peak of acute hyperalgesia, this could in turn dampen injury-induced hypersensitivity and help the acute response return to baseline. Consistent with this idea, pre-activating ILS (constitutive activation of InR) temporally shifts the dampening of nociceptive sensitivity from the recovery time point (24 h) to the peak time point (8 h). Comparing the timing and magnitude of sensitivity between *InR^RNAi^*- and *InR^CA^*-expressing larvae, the most likely time of activation of ILS following injury is during the normal recovery phase (Fig. 8).

Several models could potentially account for how InR regulates the persistence of acute sensitization. One model, consistent with our experiments with activation of InR and the timing of ILS activation, is that ILS helps shut off the acute nociceptive sensitization response. This model predicts some crosstalk between ILS and acute sensitization pathways, such as TNF, Tachykinin or Hedgehog, and/or the downstream TRP channels through which these sensitization pathways act (Babcock and Galko, 2009; Babcock et al., 2011; Im et al., 2015). Hedgehog signaling, because it is required for acute hyperalgesia (Babcock et al., 2011) and regulates metabolic effects (Rodenfels et al., 2014), seems a plausible target of ILS. A second model is that ILS effects could be more direct – insulin can affect TRPV1 sensitivity and membrane levels (Lilja et al., 2007) in some neurons. A third model postulates that ILS, once it is activated post-injury, turns on novel regulators of neuronal firing that counteract the effects of acute sensitization pathways (Augustin et al., 2017). Such regulators might include the HCN2 channel, which regulates nociceptive sensitivity during diabetic neuropathy (Tsantoulas et al., 2017) and/or GRK2, which regulates duration of acute sensitization responses (Wang et al., 2011).

Our finding that both type 1 and type 2 larval models of diabetes phenocopy sensory neuronal loss of InR suggests strongly that there is a tie to diabetes-associated pain. The persistent sensitization observed in type 1 and type 2 larval models demonstrate that, at least in principle, diabetic states can alter the behavioral response(s) mediated by sensory neurons over a highly compressed timescale and without dramatic changes to the morphology of distal terminals. This might be most relevant to the early phases of painful diabetic neuropathy that are characterized by sensory hypersensitivity, often in the absence of overt neuronal morphology changes (Wright et al., 2007). The later phase of painful diabetic neuropathy, often associated with sensory numbness, is correlated with peripheral neuronal degeneration (Kennedy et al., 1996). Is the sensory hypersensitivity phenotype observed under diabetic conditions related to hyperglycemia or insulin resistance? Tight glycemic control does not necessarily track well with pain symptoms in patients (Chan et al., 1990). Further, a number of prior studies have suggested that diabetic neuropathy can be separated from hyperglycemia (Brussee et al., 2004; Romanovsky et al., 2010). Nociceptive sensory neurons, which express the insulin receptor (Sugimoto et al., 2002, 2000), can become insulin resistant both in culture (Kim et al., 2011) and under diabetic conditions (Grote et al., 2013). These data, together with our own data supporting a nociceptor-localized role for ILS in controlling nociceptive duration, suggest that nociceptors themselves are a functionally relevant tissue for insulin action during regulation of nociception.

Our work establishes a novel genetically tractable model of neuronal InR function and diabetes-associated nociceptive changes. Such fly models can serve as hypothesis generators for complementary vertebrate approaches, as well as a platform for future gene discovery approaches (Bellen et al., 2010; Graham and Pick, 2017). One implication of our work is that diabetes-associated nociceptive changes might be more injury dependent and closely related to the acute-to-chronic switch associated with standard injury-dependent sensitization than previously appreciated. A second implication is that diabetes-associated changes in nociception could be more driven by primary changes in ILS, as opposed to secondary effects on associated tissues such as the vasculature (Powell et al., 1985). Future work on this model and testing logical hypotheses emerging from this model in vertebrate systems will determine how relevant the model is, given the added complexity of diabetes-associated sequelae and nociceptive circuitry in vertebrates.

## MATERIALS AND METHODS

### Fly stocks and genetics

Stocks were obtained from the Bloomington *Drosophila* Stock Center (NIH P40OD018537) and the Vienna *Drosophila* RNAi Center. All experimental crosses were performed at 25°C, with the exception of *InR* transheterozygotic combination (*InR^e19/93Dj4^*) (Tatar et al., 2001), which was reared at 18°C until third instar larval stage and then moved to 25°C for experiments. Flies were raised on regular corn meal media except for the type 2 diabetes experiments. A high-sugar diet (10 g/l agar, 80 g/l brewer's yeast, 20 g/l yeast extract, 20 g/l peptone, 342 g/l sucrose, 0.5 g/l MgSO_4_, 0.5 g/l CaCl_2_, 6 ml/l propionic acid, 0.1% mold inhibitor) contains 6.7 times higher sugar compared with a control diet (51 g sucrose, all other ingredients the same) (Musselman et al., 2011). *w^1118^* and/or *Gal4^109(2)80^/+* (crossed to *w^1118^*) served as control strains for behavioral analysis and staining. *InR* mutant alleles used were *InR^e19^* and *InR^93Dj4^*. Tissue-specific expression of *UAS* transgenes was controlled by *Gal4^109(2)80^* (Gao et al., 1999) or *21-7-Gal4* (Song et al., 2007) for all four classes of md neurons, *ppk1.9-Gal4* for class IV md neurons (Ainsley et al., 2003), *dilp2-Gal4* for IPCs (Rulifson et al., 2002), *hmlΔ-Gal4* (Sinenko and Mathey-Prevot, 2004) for circulating hemocytes, *Dmef2* (*Mef2*)*-Gal4* for muscle (Zars et al., 2000) and OK376-Gal4 for larval fat body (Wu et al., 2009). *UAS-Kir2.1* was used to silence IPCs and block Ilp secretion (Kim and Rulifson, 2004). *UAS-InR^DN^* (K1409A) (Wu et al., 2005), *UAS-InR^CA^* (A1325D) (Wang et al., 2008) and *UAS-InR* (Martin-Pena et al., 2006) were used to manipulate InR function, and *UAS-CaMPARI* (Fosque et al., 2015) was used to monitor Ca^2+^ levels within class IV md neurons. RNAi lines (Dietzl et al., 2007; Ni et al., 2011) used were *InR^JF01482^*, *InR^JF01183^*, *chico^JF02964^*, *Pi3K68D^GD7348^*, *Pi3K92E^GD11228^* and *Luc^JF01355^*. Table S2 lists all of the specific genotypes used in each figure panel throughout the manuscript.

### Behavioral assays

UV-induced tissue damage and thermal nociception assays were performed as described previously (Babcock et al., 2009; Chattopadhyay et al., 2012; Im et al., 2015), and a brief description follows. To induce tissue damage, early third instar larvae were etherized (Ethyl Ether Anhydrous, Thermo Fisher Scientific), immobilized and exposed to 254 nm wavelength UV at a setting of 20 mJ/cm^2^ for ∼5 s using spectrolinker XL-1000 UV crosslinker (Spectroline). During irradiation, a hand-held UV spectrophotometer (AccuMAX XS-254, Spectroline) was placed next to the specimen to read the exact UV dose – usually 11-14 mJ/cm^2^. Mock or UV-irradiated larvae were returned to fly food until thermal nociception assays were performed. For the thermal nociception assay, a metal tip of a custom-built thermal probe, the surface temperature of which is fine-tuned, touches the dorsal side of an early third instar larva in abdominal segments A3-A5. All thermal nociception assays in this paper were performed at a heat probe setting of 43°C (Babcock et al., 2009). Thermal hyperalgesia assays were performed 8 h after UV irradiation. Persistent hyperalgesia assays were performed 24 h or 42 h after UV irradiation. Aversive withdrawal behavior (corkscrew-like rolling) was scored under a dissecting stereomicroscope and the latency was recorded up to a 20 s cutoff. Behavioral assays were performed in triplicate sets of 30 or more larvae, and accumulated total percent responses were plotted as a function of latency (duration of probe contact until initiation of rolling). Statistical significance was tested using Log-rank analysis in GraphPad Prism unless noted otherwise in the figure legends.

### Live imaging and confocal microscopy

Confocal imaging of *in vivo* neuronal morphology was performed as previously described (Das et al., 2017; Turner et al., 2016). Briefly, third instar larvae were mounted on slides with 1:5 (v/v) diethyl ether:halocarbon oil and imaged on a Zeiss LSM780 confocal system. Z-stacks of class IV md neurons were obtained and neuromorphometric analyses of two-dimensional maximum projections of the z-stacks were performed using Adobe Photoshop and ImageJ (Analyze Skeleton plug in: http://imagej.net/AnalyzeSkeleton) as previously described, with modification (Iyer et al., 2013). Statistical significance was tested using one-way ANOVA with Bonferroni multiple comparison post hoc test in GraphPad Prism.

### CaMPARI analysis

CaMPARI imaging was performed as previously described (Patel and Cox, 2017; Turner et al., 2016) with the following modifications. Third instar control and *UAS-InR^RNAi^* larvae expressing *UAS- CaMPARI* were analyzed in the presence or absence of photo-converting (PC) light (440 nm excitation), UV irradiation and/or a 43°C heat probe stimulus applied as in the behavioral experiments. Z-stack images were taken using a Zeiss LSM780 confocal system at 1024×1024 pixel resolution using a Plan-Apochromat 20×/0.8 NA and 1.4 digital zoom. 3D z-stacks were transformed to 2D maximum projection images and fluorescence intensity normalized to area for F_Red_ and F_Green_ was measured using Zen blue (Lite) from Zeiss. CaMPARI responses were recorded from class IV md neurons across abdominal segments A1-A4. Identical settings for laser intensity and other image capture parameters were applied for comparison of CaMPARI responses across conditions. Statistical significance was tested using one-way ANOVA with Tukey multiple comparison post hoc test in GraphPad Prism.

## Supplementary Material

Supplementary information
