# Supplementary material for: Drosophila Insulin receptor regulates the persistence of injury-induced nociceptive sensitization
Source: Dis Model Mech. 2018 May 10;11(5):dmm034231. doi: 10.1242/dmm.034231 (PMC5992604; doi:10.1242/dmm.034231)
Supplement: Supplementary information [file dmm-11-034231-s1.pdf]

Supplementary Table 1: Gal4 Driver lines used.

Gal4	Tissues	References
Gal4$^{109(2)80}$ (md-Gal4)	multi-dendritic sensory neurons	(Gao et al., 1999)
21-7-Gal4	multi-dendritic sensory neurons	(Song et al., 2007)
ppk1.9-Gal4	class IV nociceptive md sensory neurons	(Ainsley et al., 2003)
dilp2-Gal4	Insulin Producing Cells	(Rulifson et al., 2002)
hmlΔ-Gal4	hemocytes	(Sinenko and Mathey-Prevot, 2004)
Dmef2-Gal4	larval muscles	(Zars et al., 2000)
Gal4^{OK376} (FB-Gal4)	larval fat body	(Wu et al., 2009)

Supplementary Table 2: Genotypes of larvae used in this study

Figure 1	*w^{1118}*<br>*w^{1118}; ; InR^{e19}/+*<br>*w^{1118}; ; InR^{93Dj4}/+*<br>*w^{1118}; ; $InR^{e19/93Dj4}$*
Figure 2B-2D	*w^{1118}; dilp2-Gal4/ ppkCD4tdTOM*<br>*w^{1118}; ppkCD4tdTOM/+; UAS-Kir2.1/+*<br>*w^{1118}; dilp2-Gal4/ppkCD4tdTOM; UAS-Kir2.1/+*
Figure 2E-2F	*w^{1118}; dilp2-Gal4/+*<br>*w^{1118}; ; UAS-Kir2.1/+*<br>*w^{1118}; dilp2-Gal4/+; UAS-Kir2.1/+*
Figure 3B-3D	*w^{1118}; ppkCD4tdTOM*
Figure 3E-3H	*w^{1118}*
Figure 4A-4D	*w^{1118}; $Gal4^{109(2)80}$/+*<br>*w^{1118}; ; $UAS\text{-}InR^{RNAi(JF01482)}$/+*<br>*$w^{1118}$; ; $UAS\text{-}InR^{RNAi(JF01183)}$/+*<br>*$w^{1118}$; $Gal4^{109(2)80}$/+; $UAS\text{-}InR^{RNAi(JF01482)}$/+*<br>*$w^{1118}$; $Gal4^{109(2)80}$/+; $UAS\text{-}InR^{RNAi(JF01183)}$/+*
Figure 4E-4G	*w^{1118}; ; $UAS\text{-}InR^{RNAi(JF01482)}$/ppk1.9-Gal4,UAS-mCD8::GFP*<br>*w^{1118}; ; $UAS\text{-}Luc^{RNAi}$/ppk1.9-Gal4,UAS-mCD8::GFP*
Figure 5	*w^{1118}; $Gal4^{109(2)80}$/UAS-CaMPARI; $UAS\text{-}InR^{RNAi(JF01482)}$/+*

	w^{1118}; $Gal4^{109(2)80}$/UAS-CaMPARI
Figure 6	w^{1118}; $Gal4^{109(2)80}$/+<br>w^{1118}; ; $UAS\text{-}InR^{CA(A1325D)}$/+<br>$w^{1118}$; $Gal4^{109(2)80}$/+; $UAS\text{-}InR^{CA(A1325D)}$/+
Figure 7A-7C	w^{1118}; $Gal4^{109(2)80}$/+; InR^{e19}/+<br>w^{1118}; UAS-InR/+; $InrR^{e19}$/+<br>w^{1118}; $Gal4^{109(2)80}$/UAS-InR; InR^{e19}/+
Figure 7D-7F	w^{1118}; $Gal4^{109(2)80}$/+;<br>w^{1118}; ; $UAS\text{-}InR^{CA(A1325D)}$/+<br>$w^{1118}$; $Gal4^{109(2)80}$/+; $UAS\text{-}InR^{CA(A1325D)}$/+
Figure S1	w^{1118}<br>w^{1118}; ; InR^{e19}/+<br>w^{1118}; ; InR^{93Dj4}/+<br>w^{1118}; ; $InR^{e19/93Dj4}$
Figure S2	w^{1118}; $Gal4^{OK376}$/+<br>w^{1118}; $Gal4^{OK376}$/+; $UAS\text{-}InR^{RNAi(JF01482)}$/+<br>$w^{1118}$; ; Dmef2-Gal4/+<br>$w^{1118}$; ; Dmef2-Gal4/$UAS\text{-}InR^{RNAi(JF01482)}$<br>$w^{1118}$; hmlΔ-Gal4, UAS-GFP/+<br>$w^{1118}$; hmlΔ-Gal4, UAS-GFP/+; $UAS\text{-}InR^{RNAi(JF01482)}$/+
Figure S3	w^{1118}; 21-7-Gal4/+<br>w^{1118}; 21-7-Gal4/+; $UAS\text{-}InR^{RNAi(JF01482)}$/+
Figure S4	w^{1118}; ; ppk1.9-Gal4/+<br>w^{1118}; ; ppk1.9-Gal4/$UAS\text{-}InR^{RNAi(JF01482)}$
Figure S6	w^{1118}; $Gal4^{109(2)80}$/+<br>w^{1118}; $UAS\text{-}InR^{DN(1)}$/+<br>w^{1118}; ; $UAS\text{-}InR^{DN(2)}$/+<br>w^{1118}; $Gal4^{109(2)80}$/$UAS\text{-}InR^{DN(1)}$/+<br>w^{1118}; $Gal4^{109(2)80}$/+; $UAS\text{-}InR^{DN(2)}$/+
Figure S7	w^{1118}; $Gal4^{109(2)80}$/+<br>w^{1118}; $Gal4^{109(2)80}$/+; $UAS\text{-}chico^{RNAi(JF02964)}$/+<br>$w^{1118}$; $Gal4^{109(2)80}$/+; $UAS\text{-}Pi3K68D^{RNAi(GD7348)}$/+<br>$w^{1118}$; $Gal4^{109(2)80}$/+; $UAS\text{-}Pi3K92E^{RNAi(GD11228)}$/+

References

AINSLEY, J. A., PETTUS, J. M., BOSENKO, D., GERSTEIN, C. E., ZINKEVICH, N., ANDERSON, M. G., ADAMS, C. M., WELSH, M. J. & JOHNSON, W. A. 2003. Enhanced locomotion caused by loss of the Drosophila DEG/ENaC protein Pickpocket1. *Curr Biol,* 13**,** 1557-63.

GAO, F. B., BRENMAN, J. E., JAN, L. Y. & JAN, Y. N. 1999. Genes regulating dendritic outgrowth, branching, and routing in Drosophila. *Genes Dev,* 13**,** 2549-61.

RULIFSON, E. J., KIM, S. K. & NUSSE, R. 2002. Ablation of insulin-producing neurons in flies: growth and diabetic phenotypes. *Science,* 296**,** 1118-20.

SINENKO, S. A. & MATHEY-PREVOT, B. 2004. Increased expression of Drosophila tetraspanin, Tsp68C, suppresses the abnormal proliferation of ytr-deficient and Ras/Raf-activated hemocytes. *Oncogene,* 23**,** 9120-8.

SONG, W., ONISHI, M., JAN, L. Y. & JAN, Y. N. 2007. Peripheral multidendritic sensory neurons are necessary for rhythmic locomotion behavior in Drosophila larvae. *Proc Natl Acad Sci U S A,* 104**,** 5199-204.

WU, Y., BROCK, A. R., WANG, Y., FUJITANI, K., UEDA, R. & GALKO, M. J. 2009. A blood-borne PDGF/VEGF-like ligand initiates wound-induced epidermal cell migration in Drosophila larvae. *Curr Biol,* 19**,** 1473-7.

ZARS, T., FISCHER, M., SCHULZ, R. & HEISENBERG, M. 2000. Localization of a short-term memory in Drosophila. *Science,* 288**,** 672-5.

Disease Models & Mechanisms • Supplementary information

Supplementary Figures

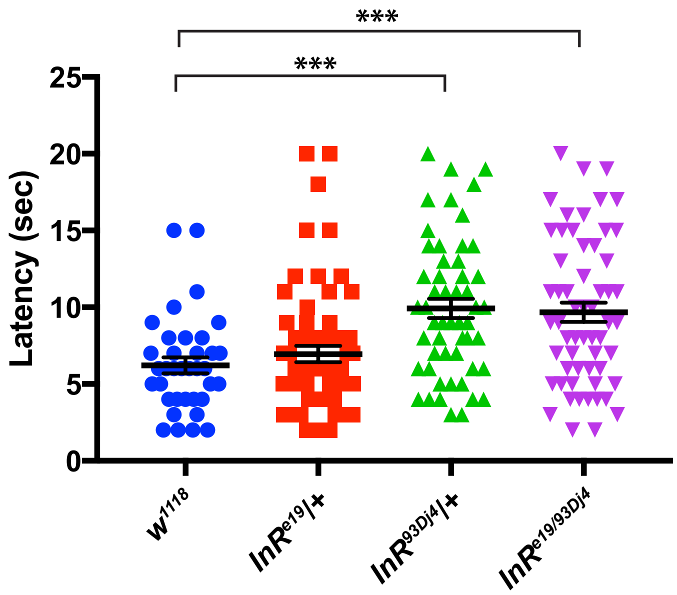

Supplementary Figure 1. InR mutant larvae show a mild increase in latency of thermal nociceptive behavior in the absence of UV injury. The data from Figure 1C (baseline, 43 °C thermal stimuli) are plotted to compare the average latency of each group. Each data point = an observed latency; middle bar = mean; error bar = standard deviation. Statistics: one-way ANOVA with Dunnett's multiple comparison post-hoc test.

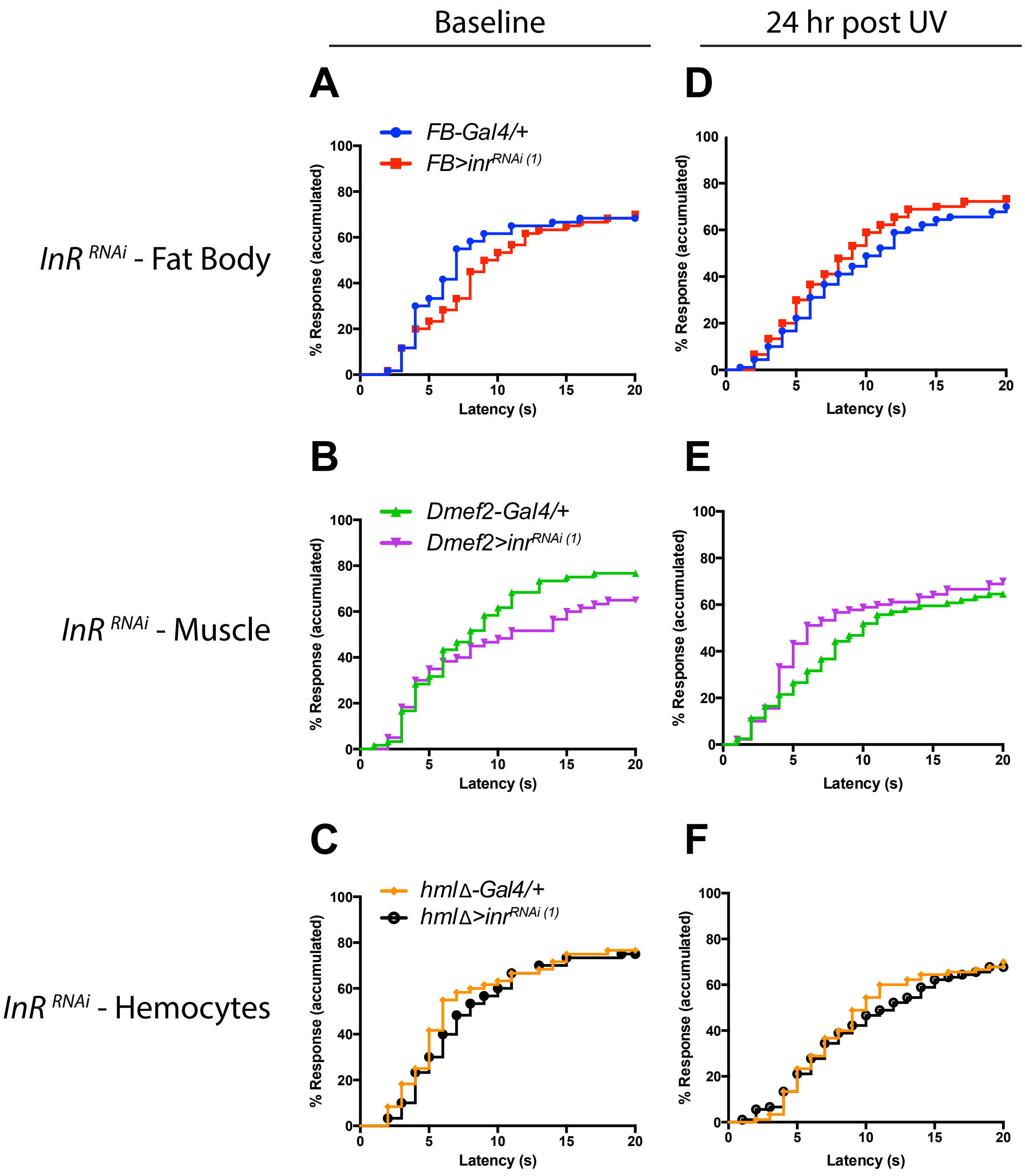

Disease Models & Mechanisms • Supplementary information

Supplementary Figure 2. Knock down of Insulin Receptor in other tissues does not cause persistent thermal hyperalgesia. Quantitation of nociceptive behavioral responses to thermal stimulation at 43 °C when $UAS\text{-}InR^{RNAi}$ is expressed in the indicated tissues. (A-C) Baseline responses without UV-induced tissue damage (n = 90). (A) Fat body, (B) Muscle, (C) Hemocytes. (D-F) Thermal sensitivity at 24 hours post UV. (D) Fat body (n = 90), (E) Muscle (n = 79 for *Dmef2-Gal4* alone control, 90 for RNAi), (F) Hemocytes (n = 90). See Table S1 for Gal4 Driver specifics.

Disease Models & Mechanisms • Supplementary information

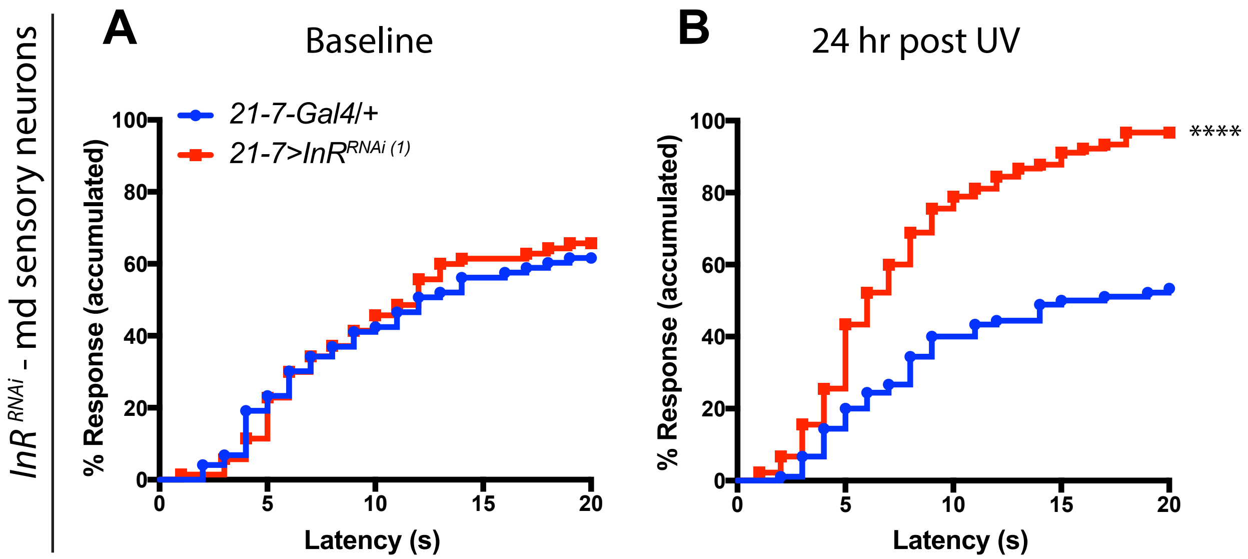

Supplementary Figure 3. Expression of *UAS-InR^{RNAi}* via an alternative md neuron-specific Gal4 driver also causes persistent thermal hyperalgesia. Quantitation of nociceptive behavioral responses to thermal stimulation at 43 °C when *UAS-InR^{RNAi}* is expressed via the md sensory neuron-specific driver *21-7-Gal4*. (A) Baseline responses without UV-induced tissue damage (n = 73 for *21-7-Gal4* alone control, 70 for RNAi). (B) Thermal sensitivity at 24 hours post UV. (n = 90).

Disease Models & Mechanisms • Supplementary information

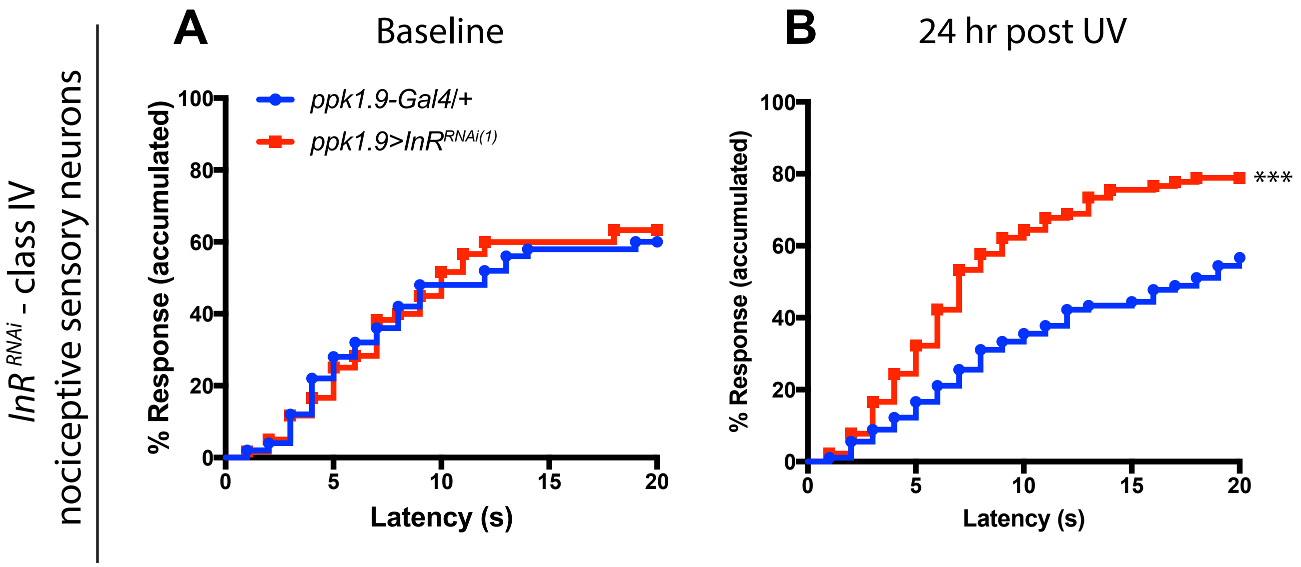

Supplementary Figure 4. Expression of *UAS-InR^{RNAi}* via a class IV md neuron-specific Gal4 driver also causes persistent thermal hyperalgesia. Quantitation of nociceptive behavioral responses to thermal stimulation at 43 °C when *UAS-InR^{RNAi}* is expressed via class IV nociceptive neuron-specific driver (*ppk1.9-Gal4*). (A) Baseline responses without UV-induced tissue damage (n = 50 for *ppk1.9-Gal4* alone control, 60 for RNAi). (B) Thermal sensitivity at 24 hours post UV. (n = 90).

Disease Models & Mechanisms • Supplementary information

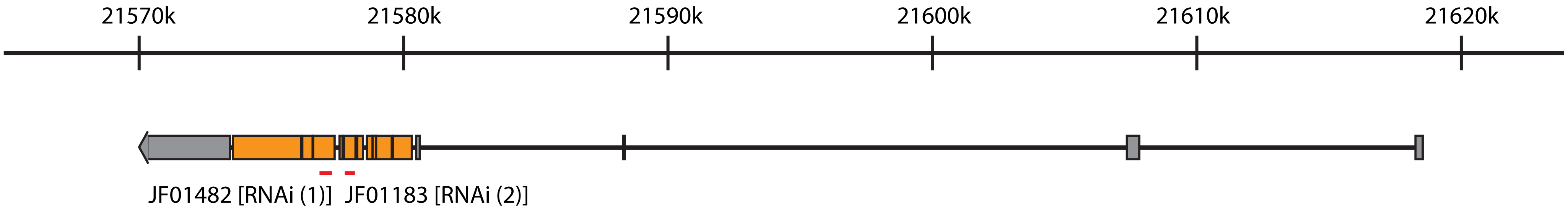

Supplementary Figure 5. Schematic of the *InR* locus and position of *UAS-RNAi* transgenes targeting *InR*. Two of non-overlapping RNAi lines targeting *InR* were used. JF01482 = *UAS-InR*$^{RNAi\ (1)}$; JF01183 = *UAS-InR*$^{RNAi\ (2)}$.

Disease Models & Mechanisms • Supplementary information

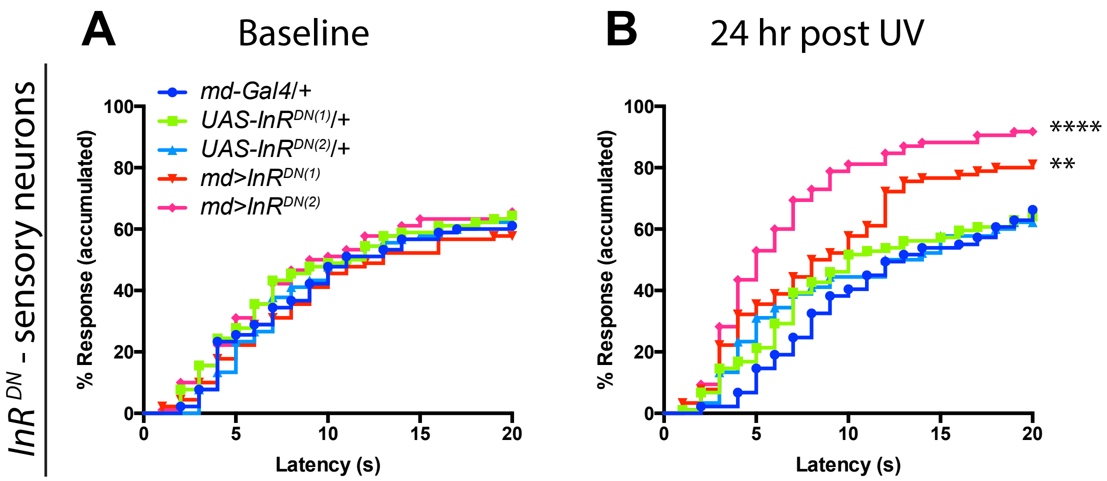

Supplementary Figure 6. Sensory neuron-specific expression of a dominant negative form of the Insulin Receptor causes persistent thermal hyperalgesia. Quantitation of nociceptive behavioral responses to thermal stimulation at 43 °C when *UAS-InR*^{DN} is expressed in md sensory neurons. (A) Baseline responses without UV-induced tissue damage (n = 90). (B) Thermal sensitivity at 24 hours post UV (n = 89 for *md-Gal4*, *UAS-InR*$^{DN(1)}$, 85 for *md> InR*$^{DN(2)}$, 90 for the rest).

Disease Models & Mechanisms • Supplementary information

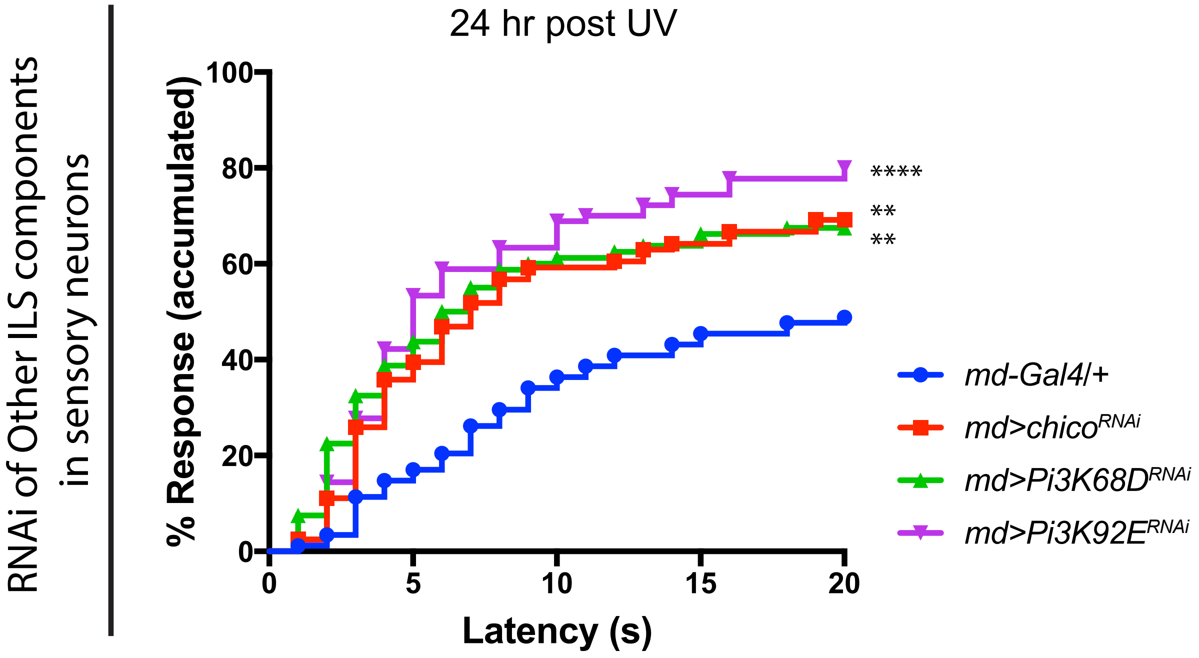

Supplementary Figure 7. md neuron-specific expression of RNAi transgenes targeting other components of ILS also causes persistent thermal hyperalgesia. Quantitation of nociceptive behavioral responses to thermal stimulation at 43 °C when *UAS-RNAi* transgenes targeting signaling components of the ILS pathway are expressed in md sensory neurons. Thermal sensitivity at 24 hours post UV (n = 88 for *md-Gal4* alone control, 81 for *chico*^{RNAi}, 80 for *Pi3K68D*^{RNAi}, 90 for *Pi3K92E*^{RNAi}).
